# Diagnosis of DVT in the emergency department: adherence to guidelines

**DOI:** 10.1186/1757-7241-20-S2-P12

**Published:** 2012-04-16

**Authors:** Christian Skjærbæk

**Affiliations:** 1 Department of Medicine, Regionshospitalet Viborg, Denmark

## Background

Patients presenting with suspected deep vein thrombosis (DVT) are common in the emergency department. Evidence based strategies for the diagnostic evaluation of these patients exists. The diagnosis is based on three criteria: a clinical pre-test probability score (Wells score), D-dimer testing, and ultrasound examination of the deep veins. The aim of our study was to evaluate the adherence to these guidelines in our emergency department.

## Methods

In a retrospective design 252 patients admitted to our department for the suspicion of DVT in 2010 were identified. All patient records were reviewed for the documentation of a pre-test clinical probability score, the result of D-dimer testing and the result of any ultrasound testing. The diagnostic work-up vas categorised as either: appropriate, inappropriate, or indeterminable due to lack of data.

## Results

Wells score was documented in 92 (37%) cases. D-dimer was measured in 252 (100%) cases and 175 (69%) patients underwent an ultrasound examination. Ultrasound examination was repeated in 18 cases.

The diagnostic work-up could be considered appropriate in 106 (42%) cases. In 53 (21%) cases the diagnostic work-up was inappropriate, in 48 (91%) cases because an ultrasound examination was not performed although indicated by the guideline and in five (9%) cases because an ultrasound examination was performed although not indicated. In 93 (37%) cases the diagnostic work-up could not be classified as appropriate or inappropriate, in all cases due to lack of documentation of Wells score.

We identified three cases were the patient was readmitted and diagnosed with DVT (n=2) or pulmonary embolism (n=1) less than three months after DVT had been ruled-out. In all three cases the appropriateness of the initial diagnostic work-up could not be determined due to lack of documentation of Wells score.

## Conclusion

We found poor adherence to diagnostic guidelines in our department. Documentation of the pre-test clinical probability is often missing and we found that under-utilisation of ultrasound is much more common than over-utilisation. Further more we found some indication that cases of DVT might have been misdiagnosed because Wells score was not applied in the diagnostic work-up.

